# Rectal Sensitivity in Diabetes Patients with Symptoms of Gastroparesis

**DOI:** 10.1155/2014/784841

**Published:** 2014-07-22

**Authors:** Eirik Søfteland, Christina Brock, Jens B. Frøkjær, Magnus Simrén, Asbjørn M. Drewes, Georg Dimcevski

**Affiliations:** ^1^Department of Medicine, Haukeland University Hospital, 5021 Bergen, Norway; ^2^Department of Clinical Medicine, University of Bergen, 5020 Bergen, Norway; ^3^Mech-Sense, Department of Gastroenterology & Hepatology, Aalborg University Hospital, 9000 Aalborg, Denmark; ^4^Mech-Sense, Department of Radiology, Aalborg University Hospital, 9000 Aalborg, Denmark; ^5^Institute of Medicine, Department of Internal Medicine & Clinical Nutrition, Sahlgrenska Academy, University of Gothenburg, 41345 Gothenburg, Sweden; ^6^Center for Sensory-Motor Interaction (SMI), Department of Health Science and Technology, Aalborg University, 9000 Aalborg, Denmark; ^7^National Centre for Ultrasound in Gastroenterology, Department of Medicine, Haukeland University Hospital, 5020 Bergen, Norway

## Abstract

In a clinical setting, diabetic autonomic complications (cardiac, gastrointestinal, urogenital, etc.) are often handled as separate entities. We investigated rectal sensitivity to heat, mechanical distension, and electrical stimulations in 20 patients with diabetes and symptoms of gastroparesis, to evaluate the extent of visceral neuronal damage. Furthermore, to evaluate the relation between the nervous structures we examined gastric emptying and cardiac autonomic function with the hypothesis being an association between these. We found that 60% of patients had delayed gastric empting. Rectal hyposensitivity was a general finding as they tolerated 67% higher thermal, 42% more mechanical, and 33% higher electrical current intensity compared to healthy controls. In patients, most heart rate variability parameters were reduced; they reported significantly more gastrointestinal symptoms and a reduced quality of life in all SF-36 domains. Shortened RR interval correlated with reduced rectal temperature sensitivity, and gastric retention rate was negatively associated with symptoms of nausea and vomiting. To conclude, in these patients with signs and symptoms of diabetic gastroparesis, rectal sensitivity was reduced, and heart rate variability was impaired. Thus, we suggest regarding diabetic autonomic neuropathy as a diffuse disorder. Symptoms of widespread autonomic dysfunction and sensory disorders should be expected and treated in these patients.

## 1. Introduction

Gastrointestinal (GI) complaints are more common in all types of diabetes mellitus (DM) patients compared to the general population [1, 2]. Symptoms such as pain, bloating, excessive fullness, vomiting and diarrhea may range from mild and intermittent to severe and life-threatening. Treatment options are limited (diet, pharmacological, and invasive procedures) and frequently incapable of adequate symptom relief [3]. Recent years have seen an improved understanding of the multiple coexisting pathophysiological mechanisms behind the symptoms. In addition to peripheral autonomic neuropathy and affection of the sensory visceral nerves, functional changes have also been detected in the brain networks encoding visceral pain [4, 5]. Mirroring this, magnetic resonance imaging techniques have revealed altered brain microstructure in the so-called “pain matrix” of the brain [6, 7]. Other mechanisms include altered elasticity of the GI wall, enterohormonal changes, anxiety/depression, exocrine pancreatic insufficiency, bacterial imbalance, autoimmunity, loss of interstitial cells of Cajal, and the direct effects of hyperglycemia on GI motility [8–10]. In line with this complex pathophysiology, the association between upper GI symptoms and gastric emptying is modest. Conversely, gastric emptying rate cannot explain the range of upper GI symptoms experienced, neither in DM patients nor in the case of idiopathic gastroparesis [11–14].

Until now, the majority of studies in this field have focused on diabetes complications of the upper GI tract. Knowledge about the extent of damage to the lower GI tract is sparse; however, in one of our recent studies we demonstrated rectal hyposensitivity in patients suffering from diabetic* sensorimotor* neuropathies [15]. A limited number of studies have examined the rectal sensitivity to distention in diabetes patients with fecal incontinence; however, none have employed multimodal sensory investigations with the possibility to investigate several nerve fibres and pathways [16, 17].

We hypothesised that DM patients with upper GI symptoms are hyposensitive in the distal GI tract and that visceral sensitivity, gastric emptying rate, cardiac autonomic function, and clinical symptoms would be associated. Thus, the main aim of this study was to examine the rectosigmoid sensitivity to multiple modalities (heat, mechanical distension, and electrical stimulations) in diabetes patients with symptoms of upper GI dysmotility. Furthermore, we aimed to characterise these patients in terms of cardiac autonomic parameters, gastric emptying rate, quality of life, and GI symptom scores.

## 2. Research Design and Methods

### 2.1. Subjects

Twenty diabetes patients were included between August 2010 and October 2011 from the outpatient clinic at Haukeland University Hospital. Inclusion criteria were upper GI symptoms refractory to treatment, type 1 or type 2 DM, and age between 18 and 65 years. All patients had previously undergone a gastroscopy in order to rule out other causes of their complaints. Major exclusion criteria were implanted gastric electrical stimulation device, nonneuropathic pain conditions, uremia, alcohol abuse, and unwillingness to cease analgesics or prokinetics prior to sensory examinations. Two patients were unable to tolerate the rectosigmoid probe, but completed the other parts of the study. As a control group, 16 healthy volunteers without GI complaints were recruited from the medical departments at Bergen and Aalborg University Hospitals. Clinical characteristics of the study population are summarized in Table 1. Oral and written consent was obtained from all participants, and the study was approved by the local ethics committees (Regional Etisk Komité Vest 2010/2562-6 and Aalborg N-20090008).

### 2.2. Gastric Emptying

Prior to the experimental rectal sensory assessments, all patients had their gastric emptying rates evaluated. Twenty spherical radiopaque markers (diameter 4 mm, density 1.27 g/mm^3^) where given together with a standardized breakfast. The number of markers still present in the stomach was determined by the help of fluoroscopy after 4, 5, and 6 hours, enabling the calculation of an average retention rate. Gastroparesis was defined as a retention rate >26% in males and >63% in females through the 4–6 hour period. The method has a sensitivity of 34% and specificity of 97% compared to scintigraphy and has been further described and validated elsewhere [11].

### 2.3. Rectal Sensory Assessment

On a separate study day, participants were instructed to fast for at least 6 hours prior to sensory examinations. In order to avoid the effect of glucose and insulin levels on GI sensations, both DM patients and healthy controls underwent a euglycemic hyperinsulinemic clamp procedure [18]. Sensory assessments were performed upon achieving the target blood glucose of 6 mM. Participants were instructed to grade the sensations using a modified Visual Analogue Scale (VAS). The scale is well known, validated, and has been employed in several studies on both upper and lower GI sensations. It runs from zero to ten, with 1 being the detection threshold, 3 being a definite moderate sensation, 5 the pain threshold, 7 moderate pain, and 10 unbearable pain [19]. After clearing the rectum with a suppository (Klyx, Ferring AS, Copenhagen, Denmark), a multimodal rectal probe (Ditens A/S, Aalborg, Denmark) was inserted through a small anoscope [20]. The probe, measuring 6.2 mm in diameter, had two channels for circulating or filling water into a noncompliant 30 *μ*m thick polyester urethane balloon placed near the tip of the probe. Two separate channels contained a thermometer and a pressure sensor. Furthermore there were two electrodes placed at the tip for electrical stimulations. Details of the probe design have been described previously [20]. Rectal thermal sensitivity was investigated by circulating 68°C water inside the rectal bag prefilled with 60 mL of 37°C water, thus enabling a gradually rising temperature. Accurate circulation flow rate (150 mL/minute) was ensured by a peristaltic pump (Ole Dich Instrument Makers, Hvidovre, Denmark). The temperature and time of circulation needed to reach sensations corresponding to 1, 3, 5, and 7 on the VAS were recorded. At VAS = 7 the heated water was immediately evacuated to minimize participant discomfort. Mechanical sensitivity was tested after emptying the rectal bag completely then infusing 37°C water at a constant rate of 200 mL/minute into the rectal bag, using the same peristaltic pump as for thermal stimulations. Three preconditioning stimulations to pain detection threshold were performed to minimise the effect of viscoelastic properties of the rectum to the mechanical distensions [20]. During the fourth stimulation the actual sensory assessment was performed, and time of filling and bag pressure needed to reach 1, 3, 5, and 7 on the VAS were recorded. When patients reached the sensation corresponding to 7 on the VAS, the pump was reversed and the bag was emptied at the same rate. Time of emptying from VAS 7 to VAS 0 was also recorded. Although the study was not principally designed to examine the rectal biomechanical properties, the rectal compliance was estimated according to a method previously described [21]. Electrical sensitivity assessment commenced after ensuring adequate mucous membrane contact by measuring the interelectrode impedance (ideally ≤ 2 kΩ). In case of higher impedance, the probe was gently manipulated and the electrical contact reassessed. The electrical stimuli were administered as 2 ms square pulses via a computer-controlled constant current stimulator (DIGITIMER Ltd., Welwyn Garden City, UK), starting at subdetection levels and increasing in increments of 1 mA. Intermittently, sham stimuli or a lower current intensity was administered, in order to limit the effect of anticipation and expectation. Participants were asked to report when the rectal sensation reached 1, 3, 5, and 7 on the VAS, and the corresponding current intensities were recorded. This multimodal sensory assessment of the rectum has been validated and described in greater detail elsewhere [20].

### 2.4. Heart Rate Variability

For evaluation of the heart rate variability—a measure of the cardiac autonomic nervous system—a 24 hour Holter ECG recording was performed in all participants (Schiller MT-200, Schiller AG, Baar, Switzerland). The following time-domain parameters where calculated: (1) RR intervals (representing the average heart rate), (2) standard deviation of normalized RR intervals (SDNN—representing the total variability), (3) standard deviation of 5-minute segments of normalized RR intervals (SDANN), (4) root mean square of the differences between successive normalized RR intervals (RMSSD—primarily representing the parasympathetic activity), and (5) the percentage of normalized RR intervals that differ more than 50% compared to the previous ones (pNN50—representing the parasympathetic dominance over the sympathetic activity) [22].

### 2.5. Questionnaires

All participants completed two questionnaires. To evaluate GI symptoms, we used the Patient Assessment of Upper Gastrointestinal Disorder Severity Symptom Index (PAGI-SYM). It consists of 20 questions, and symptoms in the preceding two weeks are graded from 0 (no symptoms) to 5 (very severe symptoms). In addition to a total score, six subscales were calculated: postprandial fullness/early satiety, nausea/vomiting, bloating, upper abdominal pain, lower abdominal pain, and heartburn/regurgitation [23]. Furthermore, the Short Form-36 (SF-36) was employed to investigate health-related quality of life. Both questionnaires have been previously translated, validated, and extensively used in Norwegian.

### 2.6. Statistics

Statistical analyses were performed in SigmaPlot 11 (Systat Software Inc., San Jose, CA, USA), using a *P* value of ≤0.05 as significance level. Results are given as means ± standard error of mean or if not normally distributed as median (interquartile (IQ) range) unless otherwise specified. To compare overall rectal sensitivities, a two-way analysis of variance (ANOVA) was employed with the factors pain modality and VAS level. When comparing the patients and healthy controls in terms of baseline characteristics, gastric emptying rate, heart rate variability, and questionnaires, one-way ANOVAs were performed. Data that were not normally distributed were compared by Kruskal-Wallis' method. Correlations between rectal sensitivity, gastric emptying, GI symptoms, and heart rate parameters were investigated by Spearman's rank order test.

## 3. Results

### 3.1. Gastric Emptying Rates

Radiopaque marker (ROM) examination was performed in all patients and was positive for gastroparesis in 60% (12 out of 20). The mean 4–6 hour ROM retention rate in women (56.4 ± 9.5%) was numerically higher than in men (40.4 ± 15.5%); however, this was not statistically significant (*P* = 0.41).

### 3.2. Rectosigmoid Sensitivity and Compliance

**Figure 1:**
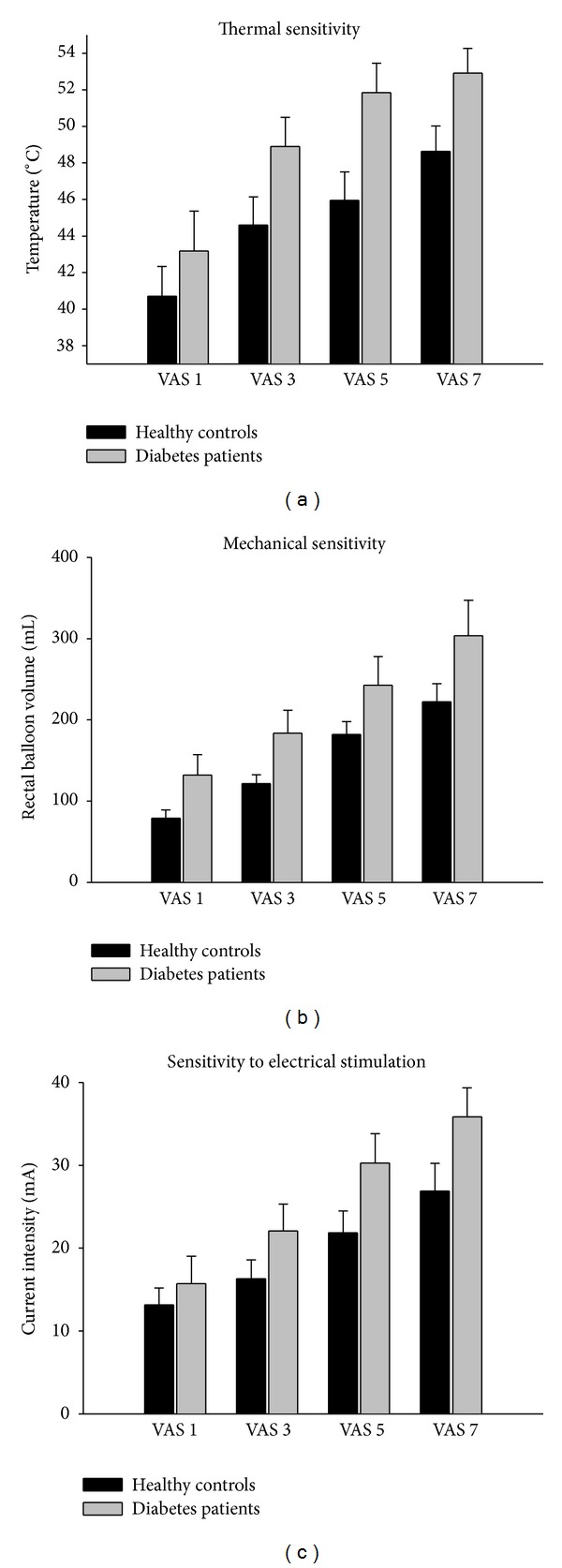
(a) The rectal sensitivity to thermal stimulation. Patients showed overall hyposensitivity to heat (*F* = 12.8, *P* < 0.001). The *Y*-axis describes the actual balloon temperature needed to induce the sensation corresponding to VAS ratings. Error bars represent SEM. (b) The rectal sensitivity to mechanical stimulation. Patients showed overall hyposensitivity to mechanical distension (*F* = 11.9, *P* < 0.001). The *Y*-axis describes the rectal balloon volumes needed to induce the corresponding VAS ratings. Error bars represent SEM. (c) The rectal sensitivity to electrical stimulation. Patients showed overall hyposensitivity to electrical stimulation (*F* = 8.8, *P* < 0.004). The *Y*-axis describes the current intensity needed to induce the corresponding VAS scores. Error bars represent SEM.

All participants were successfully clamped, and the mean blood glucose levels were similar during testing (patients 6.4 ± 0.12 and controls 6.1 ± 0.13 mmol/L, *P* = 0.12). Rectal sensitivities are summarized in Figures 1(a)–1(c). In short, diabetes patients needed significantly higher temperatures to induce the various VAS-levels compared to controls; all VAS-levels average temperature was 49.2 ± 0.80°C in patients and 45.0 ± 0.88°C in healthy controls (*F* = 12.8, *P* < 0.001). Similarly, the duration of thermal stimulation was longer in patients than in controls (74.4 ± 5.3 seconds versus 44.6 ± 5.7, *F* = 14.8, *P* < 0.001). For mechanical sensitivity there were similar results; at all VAS-levels average rectal balloon volume was 215 ± 13 mL in patients and 151 ± 13 mL in healthy controls (*F* = 11.9, *P* < 0.001). This corresponded to higher mean rectal balloon pressure at the various VAS levels in the patient cohort (22.3 ± 1.7 cm H_2_O) than in the control cohort (15.4 ± 1.7 cm H_2_O, *F* = 8.0, *P* = 0.005). However, there was no difference in compliance between patients (median 0.024 (0.020–0.048) mL/cm H_2_O) and controls (median 0.029 (0.023–0.062) mL/cm H_2_O), *P* = 0.38.

The diabetes patients were also hyposensitive to electrical stimulation and needed significantly higher current intensities to reach the predefined VAS levels. All VAS levels average current intensity was 26.0 ± 1.5 mA in patients and 19.6 ± 1.7 mA in healthy controls (*F* = 8.8, *P* < 0.004). In relative terms, diabetes patients needed 67% and 42% more time of thermal and mechanical stimulations, and 33% higher electrical current intensities to reach the same VAS levels as the healthy controls.

### 3.3. Heart Rate Variability

Technically acceptable 24-hour Holter results were obtained in 18 patients and 11 healthy volunteers. The heart rate was higher in patients (mean RR interval in patients 745 ± 106 ms compared to 820 ± 79 ms in healthy controls, *P* = 0.05). Also, most parameters of heart rate variability were reduced in patients: median SDNN 102 ms (interquartile range 71–118) versus 137 ms (118–168), *P* = 0.02; median SDANN 83 ms (60–96) versus 128 ms (98–141) *P* = 0.004. Median pNN50 was reduced in patients 2.5% (1.1–6.0) versus 13.0% (3.0–24.4) in healthy controls, *P* = 0.02. There was no difference between the two groups in terms of RMSSD, in patients 28 ms (17–46) and in controls 38 ms (23–52), *P* = 0.29.

### 3.4. Questionnaires

The patients scored significantly higher in all of the investigated aspects of upper and lower gastrointestinal symptoms (all *P* < 0.001); see Table 2. The SF-36 questionnaire revealed a strongly reduced self-reported health in the patient cohort with all SF-36 subscales reduced (all *P* < 0.05). For details, please see Table 3.

### 3.5. Clinical Correlations

According to our hypothesis, we investigated the associations between rectal sensitivity, gastric emptying, heart rate parameters, and gastrointestinal symptoms. There were no statistically significant correlations between rectosigmoid sensitivity and gastrointestinal symptoms (PAGI-SYM). The gastric retention rate was positively associated with the temperature sensitivity (*r* = 0.59, *P* = 0.02); that is, the more delayed the gastric emptying, the more increased the rectal sensitivity to temperature. There was a similar trend in mechanical pressure, but it did not reach statistical significance (*r* = 0.44, *P* = 0.08). Gastric retention was also negatively associated with symptoms of nausea and vomiting (*r* = −0.51, *P* = 0.03); that is, the more delayed the gastric emptying, the* less* the symptoms. The RR interval was negatively associated with symptoms of postprandial fullness (*r* = −0.49, *P* = 0.04) as well as the rectal temperature sensitivity (*r* = −0.63, *P* = 0.01); that is, patients with higher mean heart rate had more symptoms of fullness and reduced rectal sensitivity to heat. No other associations could be detected between these predefined parameters.

## 4. Discussion

We have shown that patients with symptoms and signs of diabetic gastroparesis had sensory deficits in the distal gastrointestinal tract, indicating a widespread nature of visceral neuropathy. Furthermore, patients had reduced heart rate variability and increased mean heart rate, a sign of extensive autonomic dysfunction in DM. Differences in rectosigmoid compliance between DM patients and controls were not detected.

Major limitations include the relatively low number of study subjects and the mixed type 1 and type 2 DM cohort. Although the two conditions share some specific pathogenetic traits (in particular hyperglycemia), the symptom presentation and gastric emptying rate may differ slightly. Type 1 DM patients have been shown to be more prone to vomiting, whereas type 2 patients have relatively more nausea. On average, gastric retention is more pronounced in type 1 DM, although the differences are subtle [24]. Method specific limitations are discussed below.

Unlike the colon, the rectum receives innervation from both visceral (sacral) and somatic (pudendal) nerves. In this study we wanted to investigate the visceral afferents specifically, and the probe was positioned at least 15 cm above the anus, thus limiting involvement of the lower somatic nerve afferents. Although rectal sensations by nature primarily deal with the feeling of fullness and the urge to defecate, we chose a multimodal approach in order to obtain a comprehensive sensory profile. Thermal stimulation has the advantage of being highly reproducible. It stimulates the mucosal receptors directly, although it is probably less physiological in nature. Mechanical stimulation, on the other hand, is more physiological but also depends on the varying elastic properties of the rectum, the muscular tone, and neuromuscular feedback loops. Finally, electrical stimulation is highly reproducible; it bypasses the peripheral receptors entirely and depolarizes the nerve endings directly [20, 25].

Gut sensitivity and motility are subject to modification by glucose and insulin levels. Both act as sensitizers to stretch in the stomach [26]. Our previous studies indicate a role for insulin, but not glucose, in the sensitivity to electrical stimulation in the esophagus [18, 27]. The anorectal region is less well investigated in this respect, and results are somewhat contradictory (increased, decreased, or no effect of hyperglycemia on sensitivity) [28–30]. To avoid any interference, we decided to use a hyperinsulinemic euglycemic clamp technique in all study subjects. This setup combined with multimodal rectal sensory assessment has been used in several previous studies and yields reproducible and physiologically meaningful results [4, 15]. In this study, no difference between diabetes patients and controls could be detected in terms of the rectosigmoid compliance; that is, the mechanosensory findings were likely unrelated to changes in rectal wall properties. Some previous studies have found reduced rectal compliance in DM, but this seems to have been strongly influenced by the actual blood glucose levels [16, 28]. In our study, euglycemia was ensured by a hyperinsulinemic clamp technique, possibly influencing our findings.

Reduced sensitivity in diabetes patients is not necessarily the product of* peripheral* nerve pathology. Altered structure and/or activity at central nervous system levels are undoubtedly present in patients with both somatic and autonomic neuropathies, as has been demonstrated by several MRI studies [7, 31–33]. Furthermore, electrophysiological studies using evoked potentials with advanced brain activity modeling have also detected abnormal brain activity patterns in patients with diabetes, autonomic neuropathy, and gastrointestinal symptoms [4, 34]. Indeed, a subset of subjects included in this study has previously been investigated using electrically induced evoked brain potentials, where functional brain changes could be detected [5]. Still, at least some degree of peripheral pathology can be argued for, as the degree of hyposensitivity varies according to the mode of stimulation; that is, the least hyposensitivity was detected in the case of electrical stimulation—bypassing the mucosal receptors, possibly indicating a noncentral involvement.

This study demonstrated that diabetes patients with symptoms of* upper* GI dysfunction have a widespread visceral hyposensitivity. Thus, it is plausible—although not yet proven—that DM affects nerves in the entire GI tract. Although clearly multifactorial, diabetic autonomic neuropathy—in particular affecting the vagal nerve—has been implicated in* upper* GI dysfunction. The afferent visceral sensory nerves innervating the* lower* GI tract travel a relatively short distance through the pelvic splanchnic nerves to the sacral parts of the spinal cord. Thus, these two visceral nerve pathways are distinct in terms of localization, length, and distribution. Our results suggest that nervous dysfunction in DM is not limited to the well-investigated vagal nerve but may also include the shorter splanchnic nerves. This implicates that small fiber diabetic neuropathy possibly is less length-dependent than large-fiber sensorimotor polyneuropathies. This, in turn, might help explain the varied clinical presentations of visceral complications in DM [35]. From a clinicians' perspective, diverse neuropathic complications should be actively considered, once a patient presents symptoms of autonomic dysfunction in any organ system.

In this explorative study, we only investigated the associations in line with our hypothesis. Gastric retention showed* positive* association with rectal sensitivity and* negative* association with the feeling of gastric fullness. That is, the more delayed the gastric emptying, the less the symptoms and the higher the rectal sensitivity to heat. Although somewhat surprising, our results are in line with a number of studies which have found very weak—if any—correlation between gastric emptying rates and symptoms of diabetic gastroparesis [36, 37]. Indeed, there is an ongoing controversy surrounding the relationship between these parameters, which strongly affects clinical intervention trials [38, 39]. Finally, the method of investigating gastric emptying—by radiopaque markers—may be less sensitive and show moderate correlation to the gold standard, scintigraphy, although the latter has shown similar poor association to upper gastrointestinal symptoms [11, 40]. Among heart rate parameters, the RR interval was associated with visceral sensitivity, that is, reduced thermal sensitivity with increasing heart rate. Increased mean heart rate is a well-known early marker of cardiac autonomic neuropathy, so any association with visceral sensitivity is in line with a pathophysiological explanation involving autonomic neuropathy [35]. A limitation of the present study in this respect is the lack of spectral frequency analysis—a method which might detect more subtle cardiac autonomic imbalance [41].

Diabetic gastrointestinal complications are challenging to investigate and diagnose. This is partly not only due to the inaccessibility of GI organs but also due to the imprecise nature of GI motility measurements and the poor correlation between symptoms, GI dysmotility, and autonomic neuropathy [42, 43]. Also, GI symptoms are so common in general that the clinical question of diabetic GI complications frequently arises. The invasive sensory investigations performed by our group in this and previous studies have demonstrated a concomitant affection of upper and lower GI tract, in conjunction with cardiac autonomic dysfunction [5]. Investigating the heart rate variability through electrocardiography or other parameters of cardiac autonomic function is easy and hazard-free for the patient. Due to multiple weak associations between various autonomic dysfunctions, these investigations are not likely to offer meaningful positive predictive information as far as diabetic gut dysfunctions are concerned. On the other hand,* normal* cardiac autonomic function might indicate that the GI signs and symptoms are* not* caused by autonomic dysfunction. This would make heart-rate variability testing a logical first step to screen diabetic from nondiabetic GI complaints. Further studies are warranted to test this hypothesis.

In conclusion, this study provided evidence of the generalized nature of diabetic autonomic neuropathy. Diabetes patients with signs and symptoms of upper GI dysfunction displayed reduced rectal sensitivity to heat and mechanical and electrical stimulation. Also, the heart rate variability was impaired. In a clinical setting, the presence of autonomic dysfunction could be regarded as a diffuse neuropathic complication.

## Figures and Tables

**Table 1:** Clinical characteristics.

Variables	Patients (*n* = 20)	Controls (*n* = 16)
Age (years)	44.5 (±9.6)	44.8 (±9.3)
Gender (male/female)	5/15	5/11
Body mass index (kg/m^2^)	26.5 (±5.1)	24.4 (±3.4)
Diabetes duration (years)	26.5 (±9.9)	—
Diabetes type (1/2)	17/3	—
HbA1c (%)	9.7 (±2.1)	5.6 (±0.2)
Smoking status (never/past/present)	10/4/5	10/6/0
Retinopathy (%)	65	—
Known neuropathy (%)	55	—
Known cardiovascular disease (%)	20	0
Creatinine level (IQ-range) (*μ*mol/L)	69.0 (58.0–104.0)	72.0 (66.5–78.0)
Beta-blocker (%)	20	0
ACEI/angiotensin receptor blocker (%)	45	6
Statin use (%)	65	6

Data are means (±SD) unless otherwise indicated.

ACEI = angiotensin converting enzyme inhibitor.

**Table 2:** PAGI-SYM scores.

		Patients	Healthy controls
Subscale item	Postprandial fullness	3.50 (2.75–4.0)	0.25 (0.0–0.44)
	Nausea/vomiting	1.33 (0.50–3.25)	0.0 (0.0-0.0)
	Bloating	3.50 (3.50–4.75)	0.0 (0.0-0.0)
	Upper abd. pain	2.76 (±0.40)	0.10 (±0.07)
	Lower abd. pain	2.00 (1.00–3.50)	0.0 (0.0-0.0)
	Heartburn/regurg.	1.14 (0.86–2.61)	0.0 (0.0-0.0)

Total score		2.25 (1.54–2.91)	0.05 (0.0–0.23)

Results of the patient assessment of upper gastrointestinal disorder severity symptom index (PAGI-SYM) questionnaire. Twenty patients and 15 healthy controls completed the questionnaire. Abd. = abdominal, regurg. = regurgitation. All *P* < 0.001.

**Table 3:** SF-36 scores.

		Patients	Healthy controls
Subscale item	Physical functioning	72.5 (40.0−85.0)	100.0 (100.0-100.0)
	Role lim. phys. (RP)	0.0 (0.0−50.0)	100.0 (100.0-100.0)
	Bodily pain	41.6 (±26.6)	88.5 (±12.6)
	General health	33.4 (±19.8)	85.2 (±16.6)
	Energy fatigue/vitality	32.5 (±18.4)	75.7 (±13.7)
	Social functioning	62.5 (37.5−75.0)	100.0 (100.0-100.0)
	Role lim. emot. (RE)	100.0 (33.3−100.0)	100.0 (100.0-100.0)
	Mental health (MH)	76.0 (68.0−80.0)	84.0 (76.0−92.0)

Summary scores	Physical comp. (PCS)	33.3 (18.8−39.2)	55.8 (54.0−57.3)
	Mental com. (MCS)	47.7 (42.9−50.0)	53.1 (48.5−55.3)

Results of the Short Form-36 questionnaire, presented as median (IQ-range) or mean (±SD). Eighteen patients and 15 healthy controls completed the questionnaire. RP = role limitations due to physical health, RE = role limitations due to emotional problems, PCS = physical component summary and MCS = mental component summary.

All *P* < 0.001 except RE: *P* = 0.02, MH: *P* = 0.04, and MCS: *P* = 0.02.

## Abbreviations

ANOVA: Analysis of variance
BMI: Body mass index
DM: Diabetes mellitus
ECG: Electrocardiography
GI: Gastrointestinal
IQ-range: Interquartile range
MRI: Magnetic resonance imaging
PAGI-SYM: Patient assessment of upper gastrointestinal disorder severity symptom index
ROM: Radiopaque marker
SEM: Standard error of the mean
SD: Standard deviation
SF-36: Short Form-36
VAS: Visual analogue scale.
